# The Complete Chloroplast Genome of *Hypoestes forskaolii* (Vahl) R.Br: Insights into Comparative and Phylogenetic Analyses within the Tribe Justiceae

**DOI:** 10.3390/genes13122259

**Published:** 2022-11-30

**Authors:** Samaila Samaila Yaradua, Kowiyou Yessoufou

**Affiliations:** 1 Department of Geography, Environmental Management and Energy Studies, APK Campus, University of Johannesburg, Johannesburg 2006, South Africa; 2 Department of Biology, Umaru Musa Yaradua University, Katsina 820102, Nigeria

**Keywords:** chloroplast genome, *Hypoestes forskaolii*, justiceae, phylogenomics

## Abstract

*Hypoestes forskaolii* is one of the most important species of the family Acanthaceae, known for its high economic and medicinal importance. It is well distributed in the Arab region as well as on the African continent. Previous studies on ethnomedicine have reported that *H*. *forskaolii* has an anti-parasitic effect as well as antimalarial and anthelmintic activities. Previous studies mainly focused on the ethnomedicinal properties, hence, there is no information on the genomic architecture and phylogenetic positions of the species within the tribe Justiceae. The tribe *Justicieae* is the most taxonomically difficult taxon in Acanthoideae due to its unresolved infratribal classification. Therefore, by sequencing the complete chloroplast genome (cp genome) of *H*. *forskaolii*, we explored the evolutionary patterns of the cp genome and reconstructed the phylogeny of Justiceae. The cp genome is quadripartite and circular in structure and has a length of 151,142 bp. There are 130 genes (86 coding for protein, 36 coding for tRNA and 8 coding for rRNA) present in the plastome. Analyses of long repeats showed only three types of repeats: forward, palindromic and reverse were present in the genome. Microsatellites analysis revealed 134 microsatellites in the cp genome with mononucleotides having the highest frequency. Comparative analyses within Justiceae showed that genomes structure and gene contents were highly conserved but there is a slight distinction in the location of the genes in the inverted repeat and single copy junctions. Additionally, it was discovered that the cp genome includes variable hotspots that can be utilized as DNA barcodes and tools for determining evolutionary relationships in the Justiceae. These regions include: *atpH*-*atpI, trnK*-*rps16, atpB-rbcL, trnT*-*trnL, psbI-trnS, matK*, *trnH*-*psbA*, and *ndhD*. The Bayesian inference phylogenetic tree showed that *H*. *forskaolii* is a sister to the *Dicliptra* clade and belongs to Diclipterinae. The result also confirms the polyphyly of *Justicia* and inclusion of Diclipterinae within justicioid. This research has revealed the phylogenetic position of *H*. *forskaolii* and also reported the resources that can be used for evolutionary and phylogenetic studies of the species and the *Justicieae*.

## 1. Introduction

*Hypoestes forskaolii* (Vahl) R.Br is a herbaceous plant and one of the most important Acanthaceaous species mainly distributed in some part of Africa and the Arab region [1]. The plant has been used in folk medicine in the treatment of various diseases like cancer, gonorrhea, heart and chest diseases [2,3]. The species is also reported to have several biological properties which includes antifungal, atrypanosomal, antileishmanial, antiplasmodial and cytotoxic properties [2,4]. A leaf decoction from the plant is used by Herdsmen to kill insect and parasite on sheep [5,6]. Fresh leaves of *H. forskaolii* are used to heal wounds and are also reported to have insecticidal activity [7]. Despite the traditional medicinal value of the plant, its complete chloroplast genome (cp genome) has not been sequenced, thus the genome evolution remains unexplored.

The tribe Justiceae is the largest tribe in Acanthaoideae (Acanthaceae) with ca. 2000 species distributed worldwide and exhibit a great diversity in morphological features. The tribe is considered the most taxonomically difficult taxon in Acanthoideae due to its unresolved tribal classification. The taxonomic problems in the tribe began after the infrafamilial classification of Acanthaceae by Lindau (in 1895). In his work, he classified *Justicia* L. and its assumed sister species in a tribe that is characterized by the presence of two stamens and two or three aperturate pollen. The problem of this classification is that he included species that don’t have this character in Justiceae and placed other species with two stamen in other tribes. Since then, several studies [8,9,10,11,12] have been conducted to address the infratribal classification. Their work was based on morphological data, but they did not analyze their data phylogenetically. However, their work contributed immensely to providing knowledge of infrafamilial and infratribal relationships within the Acanthaceae. The major setback of the Bremekamp infrafamilial classification is that he did not account for the generic composition of his tribes [11]. McDade and her colleagues (in 2000) were the first students of Acanthaceae who employed phylogenetic analysis using the *nrITS* and the *trnL-F* region from 55 species to resolve the Justiceae tribal classification. Their results recognized four lineages and these lineages are characterized by tricolporate hexapseudocolpate pollen and also provided a base for evaluating the phylogenetic relationship of the *Justicieae* species. Since then, ref. [13] worked on *Isoglossinae*, refs. [14,15,16] worked on the Tetmerium lineage. The recent study that tries to evaluate the tribal classification of Justiceae, particularly the justicoid lineages is [17]. In their study, they used six DNA regions from 178 samples of Justiceae and concluded that *Justicia* is paraphyletic and called for further investigation to address the infratribal classification of the tribe.

Understanding the evolutionary links among plant species at various taxonomic hierarchies can be achieved with the help of genetic data. This is due to the presence of various protein-coding and transfer RNA genes that play a crucial role in the cells of plants. The major role of the chloroplast organelle in a plant is the conversion of carbon dioxide and water into simple sugars [18]. The chloroplast genome (cp genome) is the most informative and conserved genome in angiosperms compared with nuclear and mitochondria. The cp genome contains sufficient information for inferring phylogenetic relationships, studies of taxa diversification and comparative analyses [19]. The cp genome of land plants usually have a circular and a quandripartite structure which include a single copy region (70–80 kb), small single copy region (15–28 kb) and a pair of inverted repeat (18–30 kb) [20]. The length of the cp genome of angiosperms ranges from 140 kb to 180 kb and they contain about 132 unique genes (ca. 86 coding for protein, 4 coding for ribosomal ribonucleic acid and 32 coding for transfer ribonucleic acid) [21]. Despite the conserved nature of the cp genome, there is some degree of variation in gene content and genome structure as well as mutations [22]. This causes the sequences of different species to diverge, which may be utilized to analyse the phylogenetic relationships of various taxonomic groups [23]. Only the chloroplast genomes of six taxa of the Justiceae have been sequenced, despite the significance of the cp genomes in contemporary systematics.

Here, we have reported and characterized the cp genome of *H. forskaolii* using the Illumina Hiseq 2500 system. We have combined the newly sequenced *H. forskaolii* with the 12 cp genomes of Justiceae sampled from the public Genbank database to explore the evolutionary circumstance of Justiceae. We have also analyzed the structure of the cp genome with other Justiceae to identify regions to be used for the species authentication and genetic diversity studies of the species including population genetics studies. We have reconstructed the phylogenetic relationship of the species using the plastome sequences to infer its phylogenetic positions and attempt to resolve the infratribal classification of Justiceae.

## 2. Materials and Methods

A typical specimen of *H. forskaolii* was collected from Batagarawa town, Katsina, Katsina State, Nigeria. The leaves from the specimen were collected and stored in a zip lock bag containing silica gel and the specimen was taken to Herbarium Center for Biodiversity and Conservation, Umaru Musa Yaradua University for identication. The specimen was authenticated by the Curator and was given accession number UMH0145. The dried leaf material was used to extract the DNA using a Qiagen genomic DNA extraction kit, following the manufacturer’s instructions.

### 2.1. Library Construction, Sequencing and Assembly

For the DNA sample preparations, 1.0 μg of DNA was used as input material. Following the manufacturer’s instructions, the NEBNext DNA Library Prep Kit was used to create the sequencing libraries, followed by addition of indices to the sample. The genomic DNA was sheared at random into segments of 350 bp, which were then prepared for further PCR enrichment and sequencing. The resulting PCR products were subject to purification (using the AMPure XP system), then the resultant libraries were measured using real-time PCR and evaluated for size distribution using an Agilent 2100 Bio analyzer. After pooling, the qualified libraries based on projected data volume and effective concentration, were fed onto Illumina sequencers. Using PRINSEQlite v0.20.4 [24], clean reads sequences (5.2 GB) were obtained from the raw data through filtering, which were then subjected to assembly using NOVOPlasty 4.2. [25] using kmer (K-mer = 39) and the cp genome of *Justicia flava* (NC 044862.1) was used as the seed and reference.

### 2.2. Gene Annotation

The online tool GeSeq [26] was used to annotate all the genes present in the cp genome of *H. forskaolii* using the plastome sequence of *Justicia flava* as reference. The sequin program was used to correct the errors in the genes that were not successfully annotated in the Geseq by adjusting the position of the codons. The cp genome architecture was drawn in OGDRAW (Organellar Genome DRAW) [26].

### 2.3. Codon Usage Analysis 

The software MEGA version 6 [27] was used to calculate the relative synonymous codon usage values (RSCU), codon usage and base composition.

### 2.4. Repeat Analysis

MicroSAtellite (MISA) (http://webblast.ipk-gatersleben.de/misa/index.php accessed on 24 November 2022) was used to identify Simple Sequence Repeats (SSRs) in the *H. forskaolii* cp genome [28]. For mononucleotides, dinucleotides, trinucleotides, and tetra, penta, and hexa nucleotides SSR motifs eight, five, four, and three repetitions units were used, respectively. The online software REPuter [29] was used to identify the types of long repeats present in the cp genome.

### 2.5. Genome Comparison

Using the annotation of *H. forskaolii* as a reference in the Shuffle-LAGAN mode [30], the cp genomes of seven species of Justiceae were compared using the tool mVISTA [31]. The online software IR scope (https://irscope.shinyapps.io/irapp/ accessed on 1 November 2022) was used to compare the border regions of the cp genome.

### 2.6. Characterization of Substitution Rate

To identify the genes that are under selection pressure, DNAsp version 6 [32] was used to examine synonymous (dS) and nonsynonymous (dN) substitution rate and dN/dS ratio.

### 2.7. Sequence Divergence

To determine sequence divergence and identify the variable regions in the cp genome, sliding window analysis was used to evaluate the diversity of nucleotide in the cp genome using DnaSP version 6 with the following parameters: 600 bp for window length and 200 bp for step size.

### 2.8. Phylogenetic Analysis

The cp genome of seventeen Acanthoideae species as well as four species, *Erythranthe lutea* (Phrymaceae), *Scrophularia dentata* (Scrophulariaceae), *Lysionotus pauciflorus* (Gesneriaceae) and *Tanaecium tetragonolobum* (Bignoniaceae) were downloaded from Genbank. The Acanthoideae species are the in group while the other four were used as the out group. Using MAFFT version 7 [33], the newly sequenced cp genome of *H. forskaolii* was aligned with the downloaded cp genomes. Using MrBayes version 3.2.6, a Bayesian inference strategy was used to reconstruct the phylogenetic tree [34]. The appropriate model was chosen using jModelTest version 3.7 [35].

## 3. Results and Discussion 

### 3.1. Characteristics of H. forskaolii Chloroplast Genome

**Figure 1:**
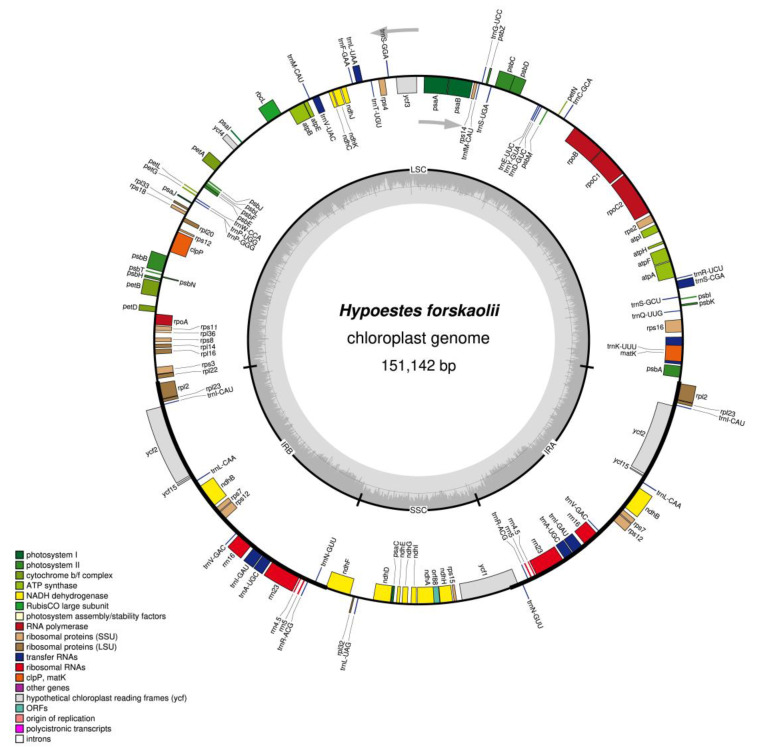
The structure of the *H. forskaolii* cp genome. Genes inside the circles are transcribed clockwise, while those outside the circles are transcribed counterclockwise. The colorful bar displays genes that are known to be functioning. The inner circle’s dark grey and light grey colors, respectively, designate the GC and AT contents.

Previous findings revealed that the cp genome of flowering plants is extremely well-preserved in both the gene content and structural organization; however, expansion and contraction in the inverted repeat and single copy junctions are among the evolutionary events leading to variations in the cp genomes [36,37]. The cp genome of *H. forskaolii* is quadripartite and circular in structure and has a total length of 151,142 bp. The cp genome consists of a pair of inverted repeats (IRb and IRa) 25,477 bp, Small Single Copy (SSC) 17,012 and Large Single Copy 83,176 bp (Figure 1). A total of 72,852 bp make up the genome’s non-coding region which is comprised of introns and intergenic spacers, and the 78,290 bp code for protein genes. The GC percentage of the LSC and SSC regions was 36.0% and 32.3%, respectively, whereas the GC content of the inverted repeats IRa and IRb was 43.4% and 43.3%, respectively (Table 1). It is discovered that the IRs have a larger proportion of GC than SSC and LSC regions.

The annotation of *H. forskaolii* cp genome revealed a total of 130 genes (86 protein-coding genes, 36 tRNAs genes and 8 rRNAs genes), 96 genes are present in the LSC (82 protein coding genes and 21 tRNA genes) and SSC (14 protein coding genes and 1 tRNA) while the remaining 17 genes (7 tRNAs, 4 rRNAs and 7 protein-coding genes) are repeated in the IRa and IRb regions (Table 2 and Figure 1). Almost all the protein-coding genes in the cp genome have ATG as their start codon and few of them have alternative start codon, GTG, ACG and ATC; this has been reported to occur in the cp genome of angiosperms [38,39,40].

Some of the coding genes present in the cp genome of *H. forskaolii* have introns. Introns are reported to occur in some of the protein-coding and tRNAs genes of flowering plants cp genomes [38,39]. Out of the 130 coding genes, 16 are characterized with one or two introns (Table 3). Among the 16 genes, six are tRNAs and 11 are protein coding genes. Ten of the intron-containing genes are located in the LSC, one gene in the SSC while the remaining five are in the inverted repeat region. ATP dependent protease subunit p gene (*clpP*) and one of the Photosysem I gene (*ycf3*) possessed two introns while the remaining 14 genes have only one intron. The tRNA, *trnK-UUU* is the gene with longest intron which is due to the inclusion of *matK* in the gene.

**Figure 2:**
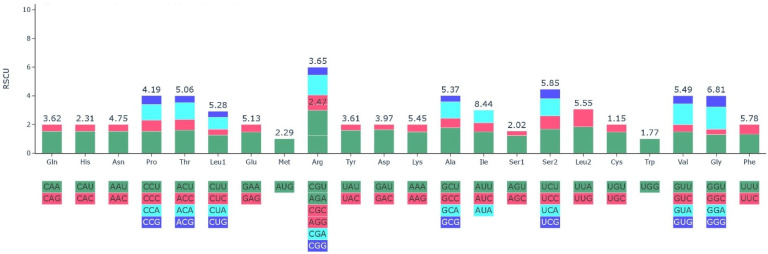
The 20 amino acids and stop codons’ relative synonymous codon usage (RSCU) in the chloroplast genome of *H. forskaolii’s* protein-coding genes.

The frequency of a codon that encodes for a specific amino acid was compared using codon usage analysis [41]. A codon is a sequence of trinucleotides that encodes for specific amino acids that are used in protein synthesis [42]. Because of bias in mutation, codon use is a factor influencing the development of the chloroplast genome [43] and it differs between species [44]. The nucleotide sequence of the protein-coding genes (78,290 bp) was used to calculate the frequency of the codons present in the cp genome. The relatively synonymous codon usage (RSCU) of the genes in the cp genome is shown in (Table 4, Figure 2). The codon usage analysis revealed that 26,095 codons encode for the genes in the cp genome. All the 20 known amino acids are encoded by 61 codons (Figure 2). Codons coding for the amino acid leucine are more prevalent in the cp genome while codons that code for Cysteine are the less common (Table 4). The Cytosine (C) and Guanine (G) endings are more prevalent than the Thymine (T) and Adenine (A) endings; the cp genomes of other angiosperms have Thymine and Adenine endings occurring more frequently [45,46,47]. The result of the analysis (Table 4) revealed that codon usage bias is low in the cp genome of *H. forskaolii*. The RSCU values for 31 codons were less than 1, and are characterized with G/C endings, whereas the RSCU values for 30 codons were greater than 1, with A/T endings. Methionine and Tryptophan are the only two amino acids without codon bias with RSCU values of 1.

### 3.2. Repeat Analyses

#### 3.2.1. Long Repeats

**Figure 3:**
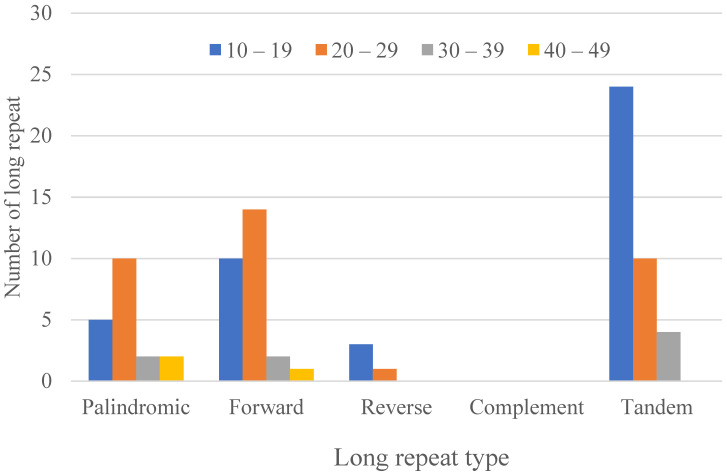
Types of tandem and long repeat distribution and their frequency in the chloroplast genomes of *H. forskaolii*.

Long repeats sequences present in the cp genome of *H. forskaolii* were identified using the program REPuter; from the results it was discovered that tandem repeats and three types of long repeats (forward, reverse and palindromic) were present in the plastome *H. forskaolii* (Figure 3). In total, there are 88 repeats in the cp genome of *H. forskaolii* (19 palindromic repeats, 27 forward repeats, 4 reverse repeats and 38 tandem repeats). Most of the palindromic and forward repeats sizes are between 20–29 bp, followed 10–19 bp. The length of repeated sequences in *H. forskaolii* cp genome ranges from 10 to 44 bp, are analogous to the lengths in other angiosperm cp genomes [48,49,50].

**Figure 4:**
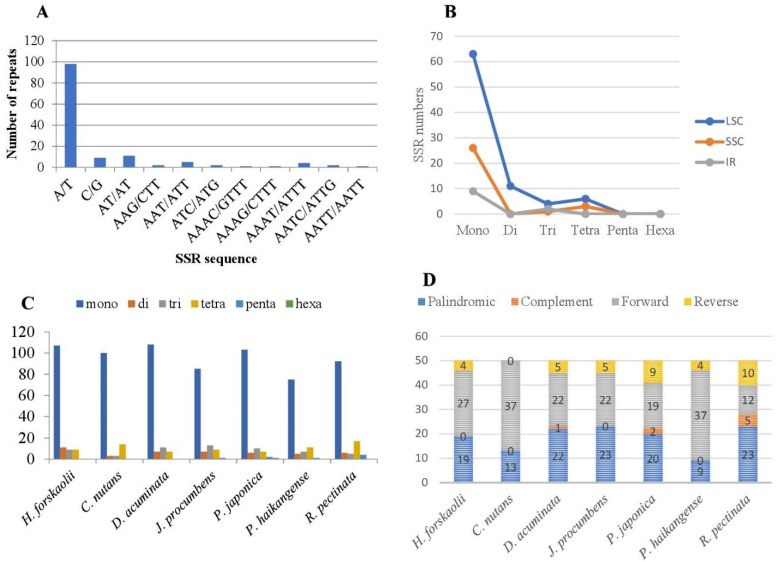
Repeat analyses of the sampled plastome of Justicae. (**A**): Frequency of different SSR motifs in different repeat types in *H. forskaolii* chloroplast genome. (**B**): Distribution of SSR in LSC, SSC, and IR regions. (**C**): Number of different SSR types in the chloroplast genome of seven Justiceae. (**D**): Number of different repeats in chloroplast genomes of seven species of Justiceae.

We compared the frequency of repeats among seven Justiceae cp genomes, and the result indicated that only three species, namely, *D. acuminata, P. japonica* and *R. pectinata* contained complement, forward, reverse and palindromic repeats (Figure 4D). *C. nutans* and *P. haikangense* have the highest frequency of forward repeats (37) while *R. pectinata* has the lowest (12). *D. acuminata* and *J. procumbes* have the same number of reverse repeats, five each. *R. pectinata* has the highest number of reverse repeats (9) while *H. forskaolii* has the lowest (4). Complement repeats are found to be the less numerous type of repeat across the genome with *D. acuminata, P. japonica* and *R. pectinata* having one, two and five, respectively.

#### 3.2.2. Simple Sequence Repeats (SSRs)

Simple sequence repeats (SSRs) are short repeats of sequences usually 1–6 bp that are very useful at evaluating genetic variation among species. These SSRs are present in cp genomes of angionsperms and are uniparently inherited. They are therefore employed as molecular markers in developmental research such as genetic heterogeneity investigations, and they also aid in the identification of species [51,52,53]. This study discovered 136 microsatellites in the cp genome of *H. forskaolii* (Table 5). Mononucleotides are the most frequent SSRs in the cp genome, constituting about 78.67%, of which majority are polythymine (41.91%) and polyadenine (30.14%); this is consistent with previous studies [53]. Among the dinucleotide only AT/AT is found in the genome. Reflecting series complementary, only two trinucleotide AAT/ATT and ATC/ATG, five tetra AAAC/GTTT, AAAG/CTTT, AAAT/ATTT, AATC/ATTG, AATT/AATT were present in the cp genome. Penta nucleotide and hexa nucleotide were not discovered in the cp genome (Figure 4A). The LSC region harbored most of the microsatellites, followed by SSC (Figure 4B).

The frequency of SSRs among the cp genomes of seven species of Justiceae was analyzed (Figure 4C); the results revealed that mononucleotides repeats are the most frequent across all the cp genomes. *D. acuminata* and *H. forskaolii* are the species with the highest frequency of mononucleotide; 108 and 107, respectively. Pentanucleotides were not present in the cp genomes of *H. forskaolii*, *D. nutans* and *D. acuminata* while hexanucleotide was only present in *P. japonica.*

### 3.3. Comparative Analysis of Justiceae Species Cp Genome

**Figure 5:**
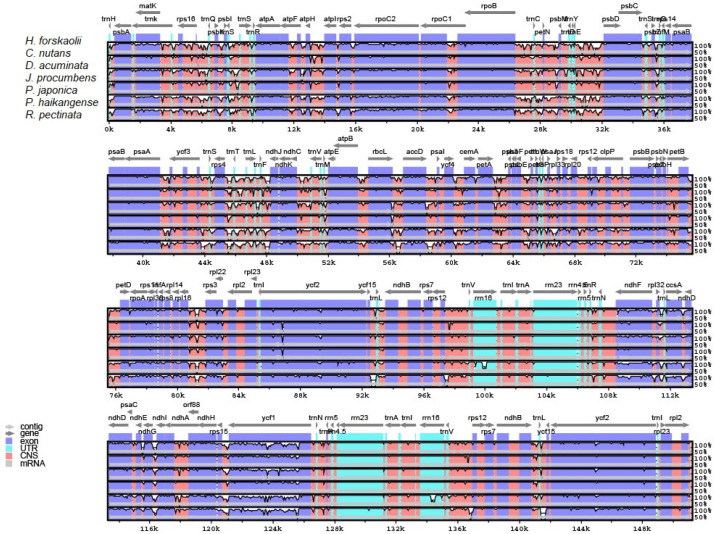
Variable regions in the cp genome of seven Justiceae species. The top arrow represents the direction of transcription; the colors blue and pink denote protein coding and conserved non-coding sequence, respectively; light green denotes tRNAs and rRNAs. The cp genome coordinates are shown by the *x*-axis, and the percentage identity ranges from 50% to 100% on the *y*-axis.

To evaluate the level of genome divergence in Justiceae, the newly sequenced cp genome *H. forskaolii* was compared with six Justiceae species cp genomes downloaded from the GenBank. The cp genomes were aligned using mVISTA with the annotation of *H. forskaolii* as a reference. The result of the anaysis revealed that the compared cp genomes are well-preserved in terms of genome structure and gene content; however, there was some level of variation. The protein-coding regions were found to be more conserved than the introns and intergenic spacers. In terms of the four regions, the IRa and IRb were more conserved than the SSC and LSC (Figure 5). This has been reported to occur in certain taxa cp genomes in earlier studies [54,55]. The most divergent non-coding regions among the seven cp genomes are *trnH*-*psbA*, *trnK*-*rps16*, *rps16-trnQ-UUG, trnE-UUC-psbD, atpH*-*atpI, trnT*-*trnL, ndhC*-*trnV*, *accD*-*psaI*, *petA*-*psbJ*, *atpB*-*rbcL*, *rps12* and *trnL*-*rpl32*. A slight sequence variation was observed in the following genes *psbM, matK*, *ycf1, trnA-UGC, ndhH*, and *rrn16*. These regions can be used as a source of potential barcode for identification/authentication of Justiceae species as well as resources for inferring phylogenetic relationships of the Acanthoideae. 

**Figure 6:**
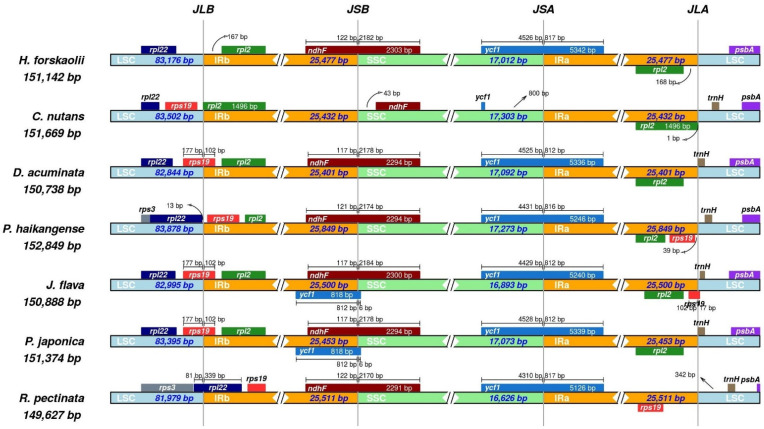
Structural variation in the junction of inverted repeat and single copy regions among the seven cp genomes of Justiceae. (JSA: Junction of the SSC and the IRA; JLB: Junction of the LSC and the IRB; JSB: Junction of the SSC and the IRB).

The structure and size of the chloroplast genome is often retained by angiosperms [53]; however, due to evolutionary processes, including genome contraction and expansion, there can be subtle variations in the size and location of the boundaries of inverted repeats and single copy regions [56,57]. We compared the JLB, JSB, JSA and JLA boundaries of the seven cp genomes of Justiceae and the results (Figure 6) showed some degree of similarity and variation among the compared cp genomes. The length of the seven cp genomes ranged from 149,627 bp (*R. pectinata)* to 152,849 bp (*P. haikangense*). Three species *J. flava*, *P. japonica* and *D. acuminata* have their JLB within the gene *rps19* with 102 bp overlapping into the IR. The JLB was bordered by *rps19* (LSC) and *rpl2* in *C. nutans* due to the contraction of the IR. In contrast, due to the IR expansion, the JLB of the *R. pectinata* was located within the *rpl22* while *P. haikangense* was between the *rpl22* (LSC) and *rps19* (IR). The JSB was found within *ndhF* with 2170–2184 bp overlapping into the IR except for the *C. nutans.* The *trnH* is located at the junction of the LSC/IRa border of all the compared cp genomes with the exception of *H. forskaolii*, which might be due to an expansion which leads to the loss of *rps19*. The cp genome of *C. nutans* varied with the other cp genomes by having the *ndhF* gene located in the SSC region. The *ycf1* pseudogene extended through the SSC and IRa with about 4300 bp in the SSC and 812 bp in the IRa in all the compared genomes except for *C. nutans*. The JSA and JSB had the most conserved borders among the compared cp genomes. The cp genome of *R. pectinata* was unique by having *rps3* in LSC/IRb border. The cp genome of *R. pectinata* had the smallest LSC region 81,979 bp while *P. haikangese* had the longest 83,878 bp. *H. forskaolii* was the only species that lost the *rps19,* which is located LSC/IR border.

### 3.4. Divergence of Protein Coding Genes Sequence 

**Figure 7:**
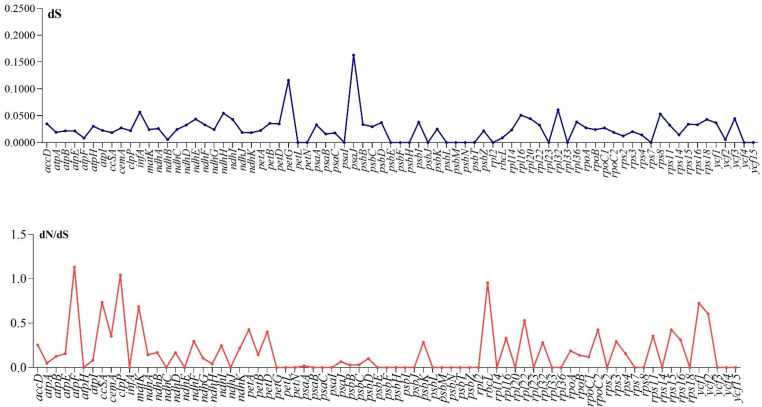
dN/dS ratio and synonymous values of the genes in the *H. forskaolii* chloroplast genome.

To determine the genes that were undergoing selective pressure in the cp genome of *H. forskaolii*, DNAsp was used to calculate the dN/dS ratio, nonsynonymous (dN) and synonymous (dS) rates. The results showed that the dN/dS ratio was less than one in almost all of the paired genes except *atpF* and *clpP* (Figure 7), indicating that most of the genes were under negative selection with the exception of *clpP* and *atpF*. The synonymous (dS) values rangee from 0.0053 to 0.1628 in all the protein-coding genes (Figure 7). Twenty-nine genes, including *ycf15, ycf3, rps18, rps14, rpl36, rpl32, psbM, psbN, psbJ, psaI, petG, ndhJ, ndhC, infA* and *atpH* showed no nonsynonymous changes occurring in the plastome of *H. forskaolii*; comparable findings were reported for other cp genomes [58,59,60].

### 3.5. Identification of Sequence Divergence 

**Figure 8:**
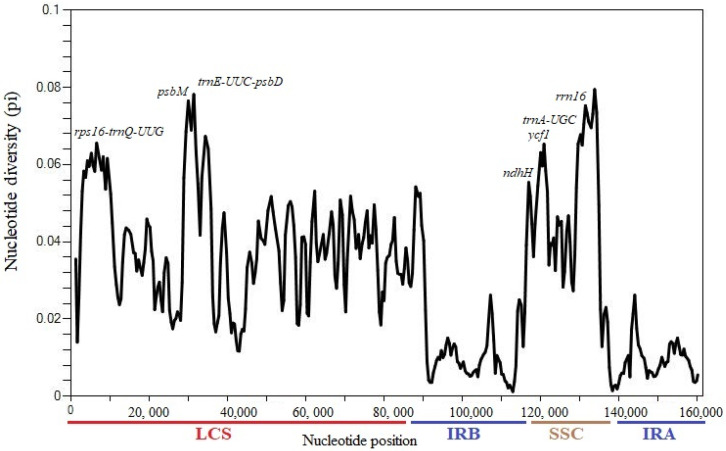
Sliding window analysis of nucleotide variability among the seven Justiceae species cp genomes (windowlength: 600 bp; step size: 200 bp).

Variable regions of the chloroplast genome are very useful in infering phylogenetic relationships and identification of species at the lowest taxonomic rank. These regions also play a vital role in providing information that helps in detecting differences between species and revealing the changes in the population structure [61,62]. The plastome sequence of *H. forskaolii* was found to be similar with that of related Justiceae species. The calculated pi values ranged from 0 to 0.79 (Figure 8), which shows slightly variation among the chloroplast genome and are relatively conserved. This pattern of variation was reported to occur in the plastomes of angiosperm [40]. Comparing the sequence divergence in the single copy regions and the inverted repeat region, the single copy region showed a higher variability than the invterted repeat. Highly variable protein-coding genes in the plastomes include *psbM, ndhH* and *ycf1*. Similarly, two intergenic spacers, *trnE-UUC-psbD* and *rps16-trnQ-UUG* were found to be highly variable. These results agree with the mVISTA divergence analysis and show that the regions could be used in the identification and authentication of Justiceae species. 

### 3.6. Phylogenetic Analysis 

**Figure 9:**
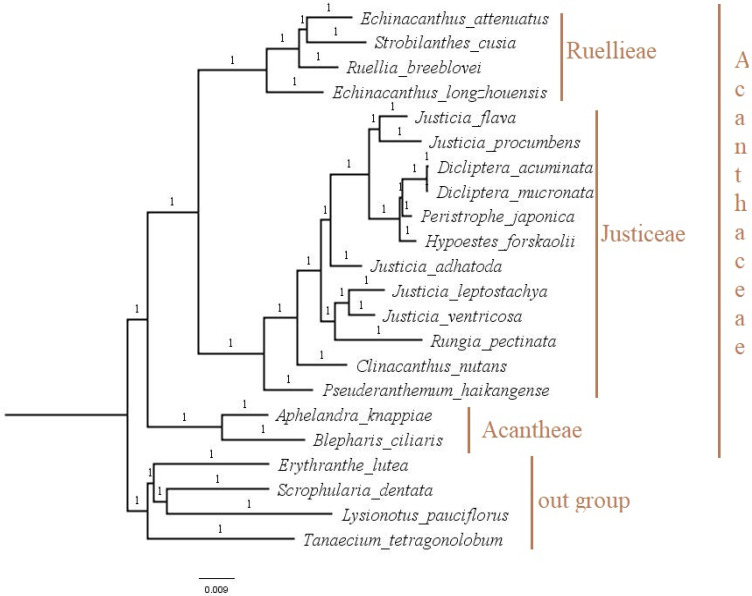
Bayesian inference phylogenetic tree reconstructed using the cp genome of 22 taxa. The branch nodes’ numbers represent the posterior probabilities values (PP).

The cp genome has been well utilized in inferring phylogenetic relationship due to its conserved nature and the presence of informative sites [63,64,65]. Various taxonomic complexes at different taxonomic levels have been resolved using phylogenetic trees reconstructed from cp genomes [66,67]. To reconstruct the phylogeny of Justiceae and infer the phylogenetic position of *H. forskaolii*, the cp genome sequences of 21 taxa were downloaded from Genbank. MAFFT version 7 was used to align the cp genome genome of *H. forskaolii* and all the downloaded cp genomes. Bayesian inference was used in reconstructing the phylogenetic tree. The results in (Figure 9), show the lineage of justiciod is strongly supported (1.0 PP) with all the twelve species belonging to the tribe Justiceae clustering in one clade (monophyletic). This is congruent with previous studies using molecular, *nrITS* and some of the chloroplast genes [0,17]. Withing the clade, there are six major sub clades, all with strongly supported posterior probability [PP] = 1.00. *H. forskaolii* forms a sister relationship with a sub clade containing *Peristrophe* and *Dicleptera* (Diclipterinae) with a strong support. A similar tree was reported by [17] and this confirmed that the species is a member of the Diclipterinae subtribe as reported by Bremekamp in his ealier classification. The core Diclipterianae (*Dicliptera, Hypoestes* and *Peristrophe*) is strongly supported in this study and members of this genera are reported to be united by some of the inflorescence part [68]. The sister relationship between *Peristrophe* and *Dicleptera* needs to be revisited by looking at their phylogenetic positions, though [69,70] suggested that the two taxa should be treated as different genera due to their differences in capsule dehiscence. The New World *Justicia* species forms a sister relationship with Diclipterinae with strong support. The phylogenetic tree clearly showed that *Justicia* is paraphyletic, as reported previously by [0,17], and there is a need to combine the phylogenetic approach with a morphological study to resolve this complexity. 

## 4. Conclusions

In this study, we have sequenced and reported on the cp genome of *H. forskaolii* to provide a valuable plastome genomic resources for the species. The plastomes have a typical gymonosperm cp genome structure they are comparable to another p genome of Acanthaceae. Simple sequence repeats used for evolutionary studies within the species were identified. The genome comparative analyses of seven Justiceae species revealed variable hotspot that could be used to develop a DNA barcode for the identification of the species. These hotspots will also be useful in phylogenetic relationship studies of the family Acanthaceae. The study has also revealed that only a few genes are under positive selection. The findings of the study have reported and confirmed the tribal position of major genera within Justiceae and has called for a further phylogenetic approach and morphological study to resolve the taxonomic complexities of the polyphyletic *Justicia* and the Justiceae.

## Figures and Tables

**Table 1:** Nucleotide composition in the *H. forskaolii* cp genome.

Region		T(U) (%)	C (%)	A (%)	G (%)	Total (bp)
cp genome		31.2	19.4	30.8	18.7	151,142
LSC		32.4	18.5	31.16	17.5	83,176
SSC		34.0	16.8	34.0	15.2	17,012
IRA		28.4	22.5	28.3	20.9	25,477
IRB		28.2	20.8	28.4	22.5	25,477
	1st Positiom	31.2	19.4	30.1	19.0	50,381
	2nd Position	31.0	19.4	31.3	18.3	50,381
	3rd Position	31.0	19.2	30.9	18.6	50,381

**Table 2:** Genes present in the chloroplast genome of *H. forskaolii*.

Category	Grpup of Genes	Name of Genes
RNA genes	ribosomal RNA genes (rRNA)	*rrn5, rrn4.5, rrn16, rrn23*
	Transfer RNA genes (tRNA)	*trnL-UAG., trnK-UUU ^+^, trnS-GCU, trnQ-UUG, trnV-UAC ^+^, trnR UCU, trnD-GUC, trnE-UUC, trnC-GCA, trnM-CAU, trnY-GUA, trnT-GGU, trnG-GCC, trnfM-CAU, trnS-UGA, trnT-UGU, trnL-UAA ^+^, trnF-GAA, trnW-CCA, trnP-UGG, trnP-GGG, trnL-CAA ^a^, trnV-GAC ^a^, trnI-GAU ^+,a^, trnA-UGC ^+,a^, trnR-ACG ^a^, trnN-GUU ^a^, trnH-GUG, trnG-UCC, trnS-GGA*
Ribosomal proteins	Small subunit of ribosome	*rps19, rps8, rps14, rps7 ^a^, rps2, rps11, rps2, rps14, rps18, rps16 ^+^, rps15, rps12 ^a^*
Transcription	Large subunit of ribosome	*rpl33, rpl16, rpl20, rpl32, rpl23 ^a^, rpl22, rpl14, rpl36, rpl2 ^+,a^*
	DNA dependent RNA polymerase	*rpoC2, rpoB rpoA, rpoC1 ^+^,*
Protein genes Other genes	Photosystem I	*psaI, psaB, psaj, psaA, psaC, ycf3 ^++^*
	Photosystem II	*psbZ, psbK, psbF, psbD, psbL, psbN, psbH, psbT, psbJ, psbB, psbE, psbM, psbC, psbI, psbA*
	Subunit of cytochrome	*petL, petB, petN, petG, petA, petD*
	Subunit of synthase	*atpH, atpF, atpE, atpB ^+^, atpA, atpI*
	Chloroplast envelope membrabe protien	*cemA*
	NADH dehydrogenase	*ndhK, ndhH, ndhI, ndhD, ndhB ^+,a^ ndhG, ndhF, ndhE, ndhC, ndhJ, ndhA^+^*
	Large subunit of rubisco	*rbcL*
	Subunit acetyl-coA carboxylase	*accD*
	ATP dependent protease subunit P	*clpP ^++^*
	Maturase	*matK*
	C-type cytochrome systhesis	*ccsA*
	Translational initiation factor	*infA*
	Component of TIC complex	*ycf1 ^a^*
	Hypothetical proteins	*ycf2 ^a^,ycf4, ycf15 ^a^*

^a^ Duplicated genes, ^+^ Gene with one intron and ^++^ Gene with two introns.

**Table 3:** Genes with introns in the *H. forskaolii* chloroplast genome and length of introns and exons.

Gene	Location	Exon I (bp)	Intron I (bp)	Exon II (bp)	Intron II (bp)	Exon III (bp)
*trnK-UUU*	LSC	36	2453	36		
*rps16*	LSC	224	912	35		
*trnS-CGA*	LSC	31	661	59		
*atpF*	LSC	470	649	140		
*rpoC1*	LSC	1635	784	433		
*ycf3*	LSC	152	710	228	683	127
*trnL-UAA*	LSC	36	513	49		
*trnV-UAC*	LSC	36	584	37		
*clpP*	LSC	226	631	299	749	69
*petB*	LSC	5	687	653		
*rpl2*	IR	435	658	397		
*ndhB*	IR	755	679	776		
*rps12*	IR	25	542	231		
*trnI-GAU*	IR	41	939	34		
*trnA-UGC*	IR	37	820	34		
*ndhA*	SSC	538	989	552		

**Table 4:** Codon usage of the *H. forskaolii* chloroplast genome.

Codon	Amino Acid	RSCU	tRNA	Codon	Amino Acid	RSCU	tRNA
UUC	Phe	0.67	*trnF-GAA*	UAU	Tyr	1.61	*trnY-GUA*
UUU	Phe	1.33		UAC	Tyr	0.39	
CUG	Leu	0.42		CAG	Gln	0.47	
UUA	Leu	1.84	*trnL-UAA*	UAA	Stop	1.55	
CUA	Leu	0.83		CAA	Gln	1.53	*trnQ-UUG*
CUU	Leu	1.27	*trnL-UAG*	CAU	His	1.54	*trnH-GUG*
CUC	Leu	0.4		CAC	His	0.46	
UUG	Leu	1.24	*trnL-CAA*	UAG	Stop	0.93	
AUG	Met	1	*trnM-CAU*	AAG	Lys	0.51	
AUU	Ile	1.5	*trnI-GAU*	AAU	Asn	1.54	*trnN-GUU*
GUC	Val	0.49		GAC	Asp	0.36	
AUA	Ile	0.89	*trnI-CAU*	AAA	Lys	1.49	*trnK-UUU*
AUC	Ile	0.61		AAC	Asn	0.46	
GUU	Val	1.5	*trnV-GAC*	GAU	Asp	1.64	*trnD-GUC*
UCU	Ser	1.68	*trnS-GGA*	UGU	Cys	1.46	*trnC-GCA*
GUA	Val	1.45		GAA	Glu	1.47	*trnE-UUC*
GUG	Val	0.56	*trnV-UAC*	GAG	Glu	0.53	
UCA	Ser	1.21		UGA	Stop	0.52	
UCC	Ser	0.92		UGC	Cys	0.54	
CCA	Pro	1.1		CGA	Arg	1.41	
UCG	Ser	0.67	*trnS-UGA*	UGG	Trp	1	*trnW-CCA*
CCU	Pro	1.51	*trnP-UGG*	CGU	Arg	1.2	*trnR-ACG*
CCC	Pro	0.78		CGC	Arg	0.41	*trnR-UCU*
GCA	Ala	1.14		GGA	Gly	1.58	
CCG	Pro	0.61		CGG	Arg	0.55	
ACU	Thr	1.61		AGA	Arg	1.78	
ACC	Thr	0.73		AGG	Arg	0.65	
GCC	Ala	0.67		GGC	Gly	0.37	
ACG	Thr	0.47	*trnT-UGU*	AGC	Ser	0.3	
GCU	Ala	1.75	*trnA-UGC*	GGU	Gly	1.27	
ACA	Thr	1.19	*trnT-GGU*	AGU	Ser	1.22	*trnS-GCU*
GCG	Ala	0.44		GGG	Gly	0.78	*trnG-UCC*

**Table 5:** Microsatellite in the chloroplast genome of *H. forskaolii*.

cpSSR ID	Repeat Motif	Length (bp)	No. of Repeats	SSR Start Position
1	(A) 8	8	23	4087; 8109; 9324; 14,945; 15,707; 18,082; 22,395; 41,631; 64,140; 80,149; 95,787; 110,230; 111,185; 112,039; 112,263; 114,062; 114,556; 118,479; 125,329; 136,542; 151,007; 151,074
2	(A) 9	9	9	7444; 11,725; 26,746; 43,488; 43,722; 66,819; 73,976; 88,658; 151,041
3	(A) 10	10	5	7650; 12,601; 14,734; 27,880; 132,799
4	(A) 12	12	1	15,729
5	(A) 13	13	1	7927
6	(A) 16	16	1	127,320
7	(A) 17	17	1	13,063
8	(C) 11	11	1	4477
9	(C) 12	12	1	131,580
10	(G) 8	8	2	4422; 57,979
11	(G) 9	9	1	5964
12	(G) 10	10	1	116,259
13	(G) 11	11	1	74,737
14	(G) 12	12	1	102,700
15	(G) 16	16	1	66,828
16	(T) 8	8	27	7431; 8012; 25,690; 30,519; 32,669; 35,551; 42,497; 45,300; 50,150; 59,760; 72,331; 74,672; 75,843; 81,161; 82,593; 82,893; 83,210; 83,277; 97,742; 109,264; 111,758; 112,374; 113,566; 123,714; 123,762; 123,859; 138,497
17	(T) 9	9	14	16,042; 17,941; 28,559; 31,561; 42,560; 65,592; 77,186; 83,242; 109,326; 115,581; 123,423; 123,827; 123,846; 145,625
18	(T) 10	10	9	11,804; 12,514; 30,933; 53,860; 68,645; 70,546; 101,483; 123,976; 125,541
19	(T) 11	11	3	59,201;123,498; 123,792;
20	(T) 12	12	2	54,320; 124,361
21	(T) 13	13	1	9522
22	(T) 16	16	1	106,956
23	(AT) 5	5	2	20,342; 46,477
24	AT (7)	7	2	7285; 13,415
25	AT (9)	9	1	75,422
26	(TA) 5	5	3	19,311; 45,521; 45,967
27	TA (6)	6	3	30,631; 45,576; 82,952
28	(ATA) 4	4	2	64,698; 149,097
29	(TTC) 4	4	1	34,430
30	(AAT) 4	4	1	63,219
31	(TTA) 4	4	1	81,350
32	(TAT) 4	4	1	85,183
33	(TGA) 4	4	1	90,439
34	(TCT) 5	5	1	124,766
35	(ATC) 4	4	1	143,840
36	(ATAA) 3	3	1	45,662
37	(TAAA) 3	3	1	66,210
38	(AAAC) 3	3	1	67,218
39	(AAAT) 3	3	1	74,480
40	(AATA) 3	3	1	113,175
41	(AATC) 3	3	1	118,310
42	(AATT) 3	3	1	122,706

## Data Availability

The complete chloroplast genome sequence of *H. forskaolii* is deposited in the GenBank with I.D no: ON398071.
